# From brick-and-mortar to livestream shopping: product information acquisition from the uncertainty reduction perspective

**DOI:** 10.1186/s40691-022-00327-3

**Published:** 2023-02-25

**Authors:** Joohye Hwang, Song-yi Youn

**Affiliations:** Department of Textile and Apparel Management, University of Missouri, Columbia, MO USA

**Keywords:** Livestream fashion shopping, Brick-and-mortar stores, Product uncertainty, Uncertainty reduction theory, Product information acquisition, Uncertainty reduction strategy

## Abstract

This study investigates how livestream fashion shopping is associated with brick-and-mortar shopping, focusing on product uncertainty, and examines livestreaming’s role in reducing product uncertainty and promoting consumers’ purchase intention by adopting the Uncertainty reduction theory (URT). The study identifies the three product information sources (i.e., product demonstration, interaction with the seller, and other viewers’ reviews) that consumers use in livestream shopping via uncertainty-reducing strategies. PLS results (n = 292) indicate that consumers who rely on salespeople’s assistance as a product information source in brick-and-mortar shopping showed a positive perception of the two product information sources—interaction with the seller and other viewers’ reviews—in livestream shopping. The seller’s product demonstration played a significant role in reducing product uncertainty and subsequently affected purchase intention, while the other two information sources (i.e., interaction with the seller and other viewers’ reviews) affected the purchase intention directly. The findings extend the URT to improve our understanding of consumer information attainment in the livestream shopping context and exemplify a promising future for livestream fashion shopping by investigating its features that can potentially substitute for the brick-and-mortar shopping experience. Future studies can include motivational factors (i.e., service and/or technical barriers) in the model.

## Introduction

Livestream shopping features product presentations with real-time social interactions between streamers and consumers (Cai & Wohn, 2019). As livestreaming services are becoming increasingly popular on social media platforms (e.g., Facebook, Instagram), retailers have adopted them as a strategy to expand their businesses (Stein, 2020). There are various types of livestream shopping available in the market, depending upon the channel origin: social media-originated (e.g., Instagram, WeChat); website-originated (e.g., Amazon, Taobao.com), and live streaming platform-originated (e.g., Live.me, Twitch) (Cai & Wohn, 2019; Kang et al*.*, 2020). Although livestreaming services’ primary purposes differ, livestream shopping is being established as a practice in the online marketplace and has been implemented explosively by retailers and brands in various forms. Indeed, with its accessibility and popularity, retailers have increased their sales by adopting livestreaming services, and the livestream shopping market is expected to generate $25 billion by the end of 2023 in the U.S. (Larson, 2021).

Fashion retailers in the U.S. have been inspired by this trend and have acquired livestreaming services in their businesses to connect with consumers (Wright, 2021). They have provided an authentic shopping experience via livestreaming platforms such as Facebook Live, Instagram Live, TikTok, Amazon Live, or their own websites. Nordstrom has launched livestream shopping for apparel and beauty products, and Macy’s introduced its first livestream shopping event in March 2021 (Larson, 2021). Moreover, luxury fashion retailers entered livestream shopping to keep consumers engaged in their business during the pandemic (Wright, 2021). For instance, Gucci launched its Gucci Live to provide customers with a near-real one-on-one shopping experience (Wright, 2021). As online channels have been reinforced under COVID-19, the digital transformation of fashion retailers toward online platforms has been accelerated to keep their businesses profitable (Youn et al., 2021). As a part of this, fashion retailers have been adopting livestream shopping to provide consumers with real-time access to their stores, products, and salespeople.

Unlike physical stores, the online environment allows fashion retailers limited formats to display tangible product features and functions, as the information is conveyed remotely (Bock et al., 2012). Specifically, even if online consumers want to obtain product information as they do in brick-and-mortar store shopping, experiential product information (i.e., fashion and apparel products), such as subjective product quality (e.g., fit/silhouette, feel, and texture) cannot be acquired in online shopping (Kim & Krishnan, 2015). In turn, product uncertainty can be aggravated because products’ features do not transfer fully to online shopping environments (Dimoka et al., 2012; Glover & Benbasat, 2010). On this account, previous scholars argued that livestream shopping would be an add-on service that online retailers can consider. Chen et al., (2019) explained that retailers collaborate with livestreaming services to transmit the streamers’ experience with authentic and synchronous features for mitigating consumer concerns related to online shopping. Even with that in mind, it has been little understood why livestream shopping experiences are associated with those at the brick-and-mortar store and how this connected mechanism mitigates consumers’ uncertainty toward fashion and apparel products. Moreover, previous literature on livestreaming services focused on entertaining expectations toward the service (Cai & Wohn, 2019; Sun et al., 2019; Wongkitrungrueng & Assarut, 2020). However, there has been a lack of scholarly discussion about utilitarian perspectives of the livestreaming services that alleviate product uncertainty and ultimately lead consumers to make their purchase decisions (e.g., Cai & Wohn, 2019; Sun et al., 2019; Wongkitrungrueng & Assarut, 2020). Therefore, it is necessary to examine the way livestreaming services can reduce product uncertainty from the understanding of shopping experiences at brick-and-mortar stores.

To fill in the gap, this study adopts the uncertainty reduction theory (URT; Berger & Calabrese, 1975), which explains information acquisition strategies for reducing perceived uncertainty. Within the theoretical framework, this study focuses on the product uncertainty (i.e., fashion and apparel products) caused by consumers’ inability to experience products in online settings compared to what they can obtain from brick-and-mortar store shopping. Finally, the purpose of the study is to (a) investigate the relationship between brick-and-mortar and livestream fashion shopping experiences for acquiring product information and (b) understand livestreaming services’ role in reducing consumers’ product uncertainty and promoting subsequent purchase intention.

## Literature review

### Livestream shopping

Livestream shopping is a type of shopping where real-time communication is delivered between the seller (i.e., streamer or host) and consumers, allowing consumers to engage with visual and interactive information (Chen, 2019). Potential customers acquire product information in various ways simultaneously during livestream shopping (Men & Zheng, 2019; Ou et al., 2014). For example, consumers interactively engage with the seller (streamer) by asking questions and commenting while receiving product information passively by watching the seller’s product demonstration (Men & Zheng, 2019). In addition, they can review the virtual communication between the seller and other audiences to learn about products simultaneously (Men & Zheng, 2019). Such information acquisition can happen concurrently, as livestream shopping allows real-time interactions between the seller and consumers (Men & Zheng, 2019).

Prior research discussed how livestreaming services were adopted successfully in online retail shopping with an emphasis on consumer motivation, social engagement, and technological benefits (Dong & Wang, 2018; Ou et al., 2014). Wongkitrungrueng and Assarut (2020) investigated consumers’ motivations to watch and stream live shows by examining the relationship between their perceived values (i.e., symbolic, utilitarian, and hedonic) and engagement through their perceived trust. They found that symbolic value influenced consumer engagement directly, while utilitarian and hedonic values affected consumer engagement through their trust in products and sellers. Other studies investigated the way social ties contribute to consumers’ purchase or repurchase intentions by examining livestreaming services’ features (i.e., interactivity and two-way communication; Dong & Wang, 2018; Ou et al., 2014). Dong and Wang (2018) emphasized livestreaming services’ technical features by adopting an information technology (IT) affordance perspective. They discussed the effects of the six identified livestream affordances (i.e., visibility, metavoicing, triggered attending, guided shopping, social connecting, and trading) on interactivity with sellers and repurchase intention. The study found that the effect was mediated through two types of social ties with sellers (i.e., strong and weak), where a strong social tie with sellers was found to be more influential than a weak social tie in enhancing repurchase behaviors. Sun et al. (2019) also investigated the influence of IT affordance (i.e., visibility, metavoicing, and guidance shopping) on consumers’ shopping engagement and their purchase intention. They categorized consumer engagement into two dimensions (i.e., social presence and immersion) and confirmed the association of IT affordance with purchase intention. Although several studies have investigated factors that influence purchase decisions, most of them focused on social relationship (e.g., level of ties) between users and sellers within a specific livestream shopping context.

### Product uncertainty

Product uncertainty refers to the perception of being uncertain about product features and functions, where a focal point in consumers’ uncertainty lies in the information asymmetry between the seller and consumer (Dimoka & Pavlou, 2008). Product uncertainty occurs due to information asymmetry, which refers to an imbalance in possession of knowledge or information between two or more parties who are engaged in the situation of information transaction (Akerlof, 1970). Dimoka et al. (2012) discussed product uncertainty as consumers’ internal experience with products in the online retail context. They explained that consumers have difficulty evaluating products because of the seller’s tendency not to disclose their products’ actual characteristics. This information asymmetry causes consumers’ inability to inspect the physical features of products, which in turn exacerbates product uncertainty (Dimoka et al., 2012). While online consumers build trust on virtual interactions with sellers to overcome the uncertainty associated with the relationship, they also perceive product uncertainty caused by their inadequate knowledge about product characteristics (Dimoka et al., 2012).

In online market settings, information asymmetry is defined as the disproportionate information shared among the participants in online shopping (Tu et al., 2017). In addition to this, product uncertainty occurs when consumers are in a situation where they have barriers or feel obstacles in evaluating products (Dimoka et al., 2012). Dimoka et al. (2012) argued that consumers’ difficulty in assessing products is attributable to sellers’ issues (i.e., inability to describe product features, unawareness of product defects, and unwillingness to reveal products’ true quality). These antecedents of product uncertainty can be discussed in the livestreaming shopping format, as product information is delivered predominantly by the seller (streamer) and cannot be obtained by the consumer directly through physical contact. Although livestream shopping offers consumers the opportunity to acquire product knowledge through its advanced technological features (e.g., high-quality visibility with audio or a live chat function) (Sun et al., 2019), it is essentially based upon remote transactions, and product uncertainty remains a hindrance in consumers’ decision-making, particularly with fashion and apparel products. Thus, this study examined the product uncertainty reduction mechanism in consumers’ internal experience with livestream shopping.

### Uncertainty Reduction Theory (URT): Product information acquisition in livestream shopping

Uncertainty reduction theory (URT) explains that people need information when they first interact with an unknown party to mitigate uncertainty and obtain predictable results (Berger & Calabrese, 1975). The URT was developed originally in an in-person context and was extended further to the online context (e.g., social media, online shopping). In the social media context, the URT was adopted to examine ways to reduce the level of perceived uncertainty toward new acquaintances on social network platforms (Antheunis et al., 2010). In the online shopping context, the role of acquiring product information in reducing product uncertainty has been explored within the URT (Tang & Lin, 2019), where acquiring product information resolves information asymmetry between the seller and consumer (Dimoka & Pavlou, 2008). These studies that applied URT in an online context suggested that people will use various sources when collecting information to reduce their uncertainty (Whaley & Samter, 2013).

Berger and Burgoon (1995) introduced three strategies—passive, interactive, and active— that people use to acquire information about the target person or object to reduce uncertainty depending upon the sources of information and the way information is acquired. Passive strategy refers to an unobtrusive uncertainty-reducing strategy in obtaining product information by observing *product demonstrations* on the website without drawing attention from others (Tang & Lin, 2019). Interactive strategy refers to a two-way communicative uncertainty-reducing strategy in obtaining product information directly through *interaction with the seller* (Berger & Burgoon, 1995; Tang & Lin, 2019). Active strategy refers to a proactive way of obtaining relevant product information through indirect sources, such as *others’ reviews* about the product, while avoiding direct communication with the seller to reduce product uncertainty (Berger & Richard, 1975; Tang & Lin, 2019).

Previous studies focused on consumers’ perceived uncertainty toward products and investigated the way uncertainty levels mediate the relationship between the three types of information sources and willingness to purchase products through the tree uncertainty reducing strategies within an online environment (e.g., shopping cart abandonment on the website, communication over social media) (Dimoka et al., 2012; Kim & Krishnan, 2015; Tang & Lin, 2019). Their findings implied that livestream shoppers may also similarly experience product uncertainty that is caused by insufficient product information and information asymmetry. Livestream shopping is where consumers can experience the three ways of uncertainty reducing strategies in parallel while shopping online. Thus, this study proposes the following three major information sources about products that consumers obtain through each corresponding strategy in the context of livestream shopping: product demonstration, interaction with the seller, and other viewers’ reviews.

#### Product demonstration

Product demonstration is a source that livestream shoppers obtain product information *passively* by observation to reduce product uncertainty (Tang & Lin, 2019). Livestream shopping gives consumers more detailed information about products through its high visibility features that provide video files and sounds that complement product presentations (Chen & Lin, 2018). Consumers seek such detailed product information and product descriptions (i.e., how to display or use the product) in online shopping contexts, as they have limited ability to assess products physically. While traditional online shopping platforms deliver typed or written product information with product pictures, livestream shopping provides streamers’ product demonstrations that show how to use the product’s features; further, they try on the items in place of the viewers (Bründl et al., 2017). This visual information that streamers convey offers consumers the opportunity to experience products vicariously by giving them a sense of social presence (Li et al., 2020). Because of the vividness of the livestreaming shopping experience (Yim et al., 2017), consumers are attracted more readily to the streamer’s demonstration of products, as it allows them to visualize products based upon their detailed descriptions and explanations.

#### Interaction with the seller

Interaction with the seller is a source of product information anchored in the direct two-way *interactive* communication with the sellers that can reduce product uncertainty in livestream shopping (Tang & Lin, 2019). As a computer-mediated communication tool, livestreaming services allow consumers to obtain product information by interacting and communicating with sellers directly in real time (Ou et al., 2014). Through real-time communication, consumers can acquire product knowledge that could resolve information asymmetry between the seller and consumers, so that both parties can reach a mutually satisfactory outcome (Ou et al., 2014). Such interactive communication enhances trust between sellers and consumers by providing a suitable environment to acquire product information and reducing the product uncertainty (Ou et al., 2014; Tang & Lin, 2019). In a livestreaming shopping environment, consumers can ask questions and obtain the answers directly from the sellers’ body, facial, and verbal cues (Li et al., 2020), similar to the way they experience this in physical shopping environments. By engaging and communicating with sellers virtually, consumers can acquire the information related to product functions, features, or performance that they need.

#### Other viewers’ reviews

Other viewers’ reviews is an indirect source of product-relevant information that livestream shoppers *actively* obtain beyond the scope of direct sources of information from the seller, including information obtained from other viewers’ reviews or interactions (Berger & Richard, 1975; Tang & Lin, 2019). In the livestream shopping format, shoppers can learn about the target product using other viewers’ comments or reviews while avoiding direct communication with the seller/streamer (Men & Zheng, 2019; Tang & Lin, 2019). User-generated comments and reviews during livestreaming services help consumers indirectly learn about the product in which they are interested (Li et al., 2020). Previous studies described it as an opportunity in which livestream shoppers are exposed to electronic word of mouth and observational learning (Chen et al., 2011). Men and Zheng (2019) confirmed the effects of both sources in livestream shopping on perceived seller credibility and their intention to purchase. Compared to consumer-generated information shared in the traditional e-commerce context, the unique characteristic of sharing information via livestreaming is that all of the comments or feedback are offered in real-time (Li et al., 2020). Consumers can promptly observe other consumers’ feedback or response toward the product or service in real time. This instant exposure encourages consumers to purchase by giving them a feeling of pressure to buy the product while it is available (Men & Zheng, 2019).

### Brick-and-mortar store shopping: product information acquisition

In brick-and-mortar stores, consumers have the ability to have concrete fashion product experiences through two major sources of information: salespeople’s assistance and consumers’ need for touch (Haas & Kenning, 2014; Lee et al., 2017; Peck & Childers, 2003a). Those two sources were investigated to understand how consumers’ expectations of obtaining product information in brick-and-mortar stores can be compensated within the online shopping context (Lee et al., 2017). Although livestream shopping is a type of online shopping, it can provide consumers with a vicarious experience that traditional online shopping cannot offer. Thus, this study extends to the livestream shopping context to examine if two sources can be substituted with the information sources of livestream shopping.

#### Salespeople’s assistance

Receiving assistance or support from salespeople is one of the most prominent sources fashion consumers use to obtain product information in brick-and-mortar stores (Reynolds & Beatty, 1999). Rippé et al. (2018) pointed out the essential role of trust in salespeople in retailing contexts. They found that salespeople’s selling efforts and input have significantly influenced consumers’ purchasing process by focusing on the social aspect of shopping (Rippé et al., 2018). Salespeople help consumers to identify their purchase demands and recommend ideal products that meet their needs (Reynolds & Beatty, 1999). Haas and Kenning (2014) investigated situational and individual motivations that explain shoppers’ consultation with salespeople. They found that shoppers with utilitarian purposes tend to obtain benefits from salespeople in the physical stores for two reasons: the need to mitigate purchase uncertainty and the desire for efficiency (Haas & Kenning, 2014). They found that insufficient information related to attributes of the product causes consumers to experience uncertainty, where salespeople’s assistance satisfies utilitarian motives for shopping (i.e., need for product knowledge and shopping efficiency). This ultimately reduces consumers’ time spent in stores (Haas & Kenning, 2014; Reynolds & Beatty, 1999).

#### Need for touch

It is a unique motivational source that consumers obtain product information in brick-and-mortar store shopping—the actual and physical experience with fashion products. This concept can be discussed by applying utilitarian and hedonic perspectives. Fashion shopping behavior is highly involved with multi-sensory experiences that allow consumers to obtain and evaluate fashion products (Workman, 2010). Different from online shopping channels, physical stores offer consumers access to actual products, which satisfies both utilitarian and hedonic motivations (Workman, 2010). Utilitarian motivated fashion consumers experience the product properties of texture, fabric feel, or weight, while hedonically motivated consumers enjoy examining and touching products when making their decisions (Haas & Kenning, 2014). These motivations are closely related to the psychological touch and goal-directed touch that focuses on a product’s properties (Peck & Childers, 2003a, 2003b). From a utilitarian perspective, Peck and Childers (2003b) explained that the primary aspect of physical fashion stores is that consumers can acquire product information and evaluate products through goal-directed instrumental touch. They suggested that this elicits consumers’ confidence in purchases by providing tangible information, which prevents consumers from experiencing the reluctance they do when shopping online.

Due to the important role of the need for touch in consumers’ decision-making process, recent studies have explored the role of haptic information in the digital and online context. De Canio and Fuentes-Blasco (2021) examined the mechanism of how haptic information mitigates and influences consumers’ intention of channel stickiness and impulse buying. They found that consumers’ channel preferences vary depending on their haptic traits (i.e., autotelic and instrumental), and consumers with a strong haptic trait preferred physical and mobile shopping channels. This indicates the importance of the need for touch aspects in virtual shopping (De Canio & Fuentes-Blasco, 2021). Another study focused on whether augmented reality (AR) technology-based content in online shopping contexts can potentially substitute for the physical shopping experience (Gatter et al., 2022). They found that an AR feature in online shopping provides utilitarian and hedonic benefits to consumers, leading them to purchase products. There was a significant association between consumers with a high autotelic need for touch and their utilitarian and hedonic benefits. Particularly, their finding implies consumers with a high need for touch expected utilitarian benefits rather than hedonic benefits, indicating that virtual content could substitute the physical shopping experience of consumers with a high utilitarian motivation (Gatter et al., 2022).

## Hypotheses development

### Product information acquisition: influence of brick-and-mortar stores on livestream shopping

In this study, we suggest that for fashion products, consumers’ ways of obtaining product information in brick-and-mortar stores would be positively associated with three ways of information acquiring communication in livestream shopping: (a) product demonstration, (b) interaction with the seller, and (c) other viewers’ reviews. Consumers who shop at physical stores have advantages in accessing and evaluating products, as the evaluation process is based upon their desire to receive salespeople’s assistance and experience with the touch and feel of products (Workman, 2010).

In relation to the experience of receiving salespeople’s assistance at a physical store, previous literature focusing on livestream has pointed out that viewers have a near-real experience through two-way interactions during live streaming (Lu et al., 2019). In a livestream shopping context, viewers (i.e., consumers) can obtain product information and details about fashion products’ texture, feel, and fit/silhouette by interacting and engaging with sellers and other viewers’ comments (Ou et al., 2014). The results imply that real-time interactions with sellers through livestream chat provide a customized experience that is comparable with the one that consumers have in physical stores. Through interactions in the live-streaming shopping environment, consumers can ask question, and they can obtain the instant and personalized product information which best fits their needs directly from the sellers, similar to how they are experienced in a physical shopping environment (Xu et al., 2020). Similarly, other viewers’ comments and reviews in real-time also contribute to acquiring product details in a viewer-generated information format (Men & Zheng, 2019).

Regarding experience with product evaluation through touch and feel in a physical store, live streaming services can provide viewers with IT-mediated product presentation and a vicarious experience with the products (Chen et al., 2019). As the fashion product is a symbolic lifestyle category associated with a high-involved consumer experience, traditional online shopping is insufficient to transmit the product experience (e.g., feel and touch). However, through livestream shopping, streamers try on fashion items and deliver product demonstrations, and viewers experience the product features vicariously (Chen et al., 2019). IT-mediated live streaming enables streamers to present products’ experience attributes (i.e., visual, tactile, and intangible attributes). As a response to consumers’ requests, streamers can show a product’s visual features from various angles synchronously. They can also transmit their tactile experience (e.g., smell, touch, and feel) by providing descriptions of tactile attributes that would be the same as viewers’ experiences based on the shared similar preferences and provide professional interpretation of intangible attributes (Chen et al., 2019). Through this vicarious product experience demonstrability, consumers can have a near-real shopping experience by obtaining product information and evaluating products as they do in a physical store. Thus, we suggest the following hypotheses.

- H1. Consumers who tend to rely on salespeople’s assistance to obtain product information at brick-and-mortar stores will positively perceive product information through (a) product demonstration, (b) interaction with the seller, and (c) other viewers’ reviews in livestream fashion shopping.
- H2. Consumers who tend to rely on the need for touch to obtain product information at brick-and-mortar stores will positively perceive the three sources of product information—(a) product demonstration, (b) interaction with the seller, and (c) other viewers’ reviews—in livestream fashion shopping.

### Effect of product information sources in brick-and-mortar stores on product uncertainty

As discussed earlier, physical stores’ accessibility to products and salespeople’s assistance offers consumers an experiential opportunity to evaluate products based on their subjective experience (Haas & Kenning, 2014; Peck & Childers, 2003b; Workman, 2010). The need for touch has a crucial role in obtaining or evaluating product information by increasing familiarity and decreasing uncertainty about products (Peck & Childers, 2003a). Salespeople’s assistance is based on real-time two-way communication between a salesperson and a consumer. It provides an opportunity for consumers to obtain information about product quality and features (Haas & Kenning, 2014). As this customized experience in obtaining product knowledge and navigating products can alleviate consumers’ uncertainty related to purchasing products, we propose the following hypothesis.

- H3. The two product information sources—(a) salespeople’s assistance and (b) need for touch—in brick-and-mortar store shopping will reduce consumers’ product uncertainty.

### Effect of product information sources in livestream shopping on product uncertainty

Although livestream shopping is equipped with authentic technical features (i.e., real-time interaction), product uncertainty remains, as the product information is delivered virtually. Compared to traditional retail settings, in the online shopping environment, it is not easy for consumers to perceive subtle information that could be obtained from social cues, such as personal interactions or non-verbal cues (i.e., body language, facial expression), which aggravates uncertainty (Dimoka et al., 2012). Product uncertainty occurs in the context of online shopping when the physical separation between consumers and products prevents consumers from evaluating them (Koppius et al., 2004; Tang & Lin, 2019). Previous researchers identified that interpersonal communication (i.e., passive, interactive, active) reduced product uncertainty in virtual shopping environments (Tang & Lin, 2019). Therefore, we suggest that information sources of seller’s product demonstration, interaction with sellers, and other viewers’ reviews may reduce product uncertainty in the livestream shopping context.

- H4. The three product information sources—(a) product demonstration, (b) interaction with the seller, and (c) other viewers’ reviews—in livestream fashion shopping will reduce consumers’ product uncertainty.

### Effect of livestream shopping and product uncertainty on purchase intention

Further, we suggest that product information obtained from the three product information sources can directly influence consumers’ purchase intention. Previous researchers have found a direct positive relationship between product knowledge and purchase decisions together with other factors (i.e., product price and product ratings; Chang & Wildt, 1994; Sun et al., 2020). This shows that when consumers receive product information, they are more likely to purchase the product online (Chang & Wildt, 1994; Sun et al., 2020). Conversely, product uncertainty in online shopping contexts has a direct negative relationship with purchase intention. Consumers believe that they are likely to make an improper choice and have less desirable shopping outcomes when they feel uncertain about a product, as the perception of uncertainty has been identified as one of the major factors that undermines and hampers online purchase decisions (Park et al., 2005; Tang & Lin, 2019). Prior studies on this topic have demonstrated that product uncertainty reduces consumers’ intention to purchase products (Chang & Wildt, 1994; Tang & Lin, 2019). Thus, we pose the following hypotheses.

- H5. The three product information sources—(a) product demonstration, (b) interaction with the seller, and (c) other viewers’ reviews—in livestream fashion shopping will positively influence consumers’ purchase intention.
- H6. Product uncertainty will negatively influence fashion consumers’ purchase intention in live stream shopping.

### Effects of product information sources of livestream shopping on purchase intention mediated through product uncertainty reduction

**Fig. 1:**
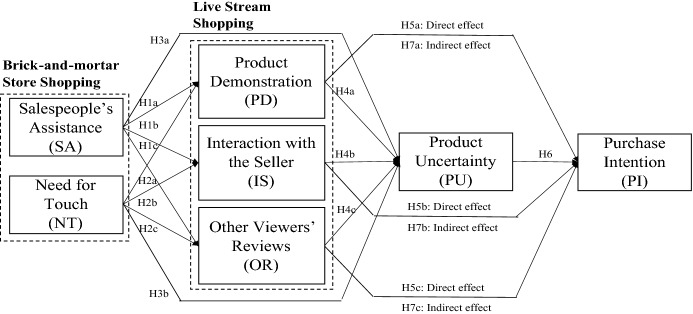
Research model

Previous researchers have investigated the mediating role of product uncertainty that affects consumers’ purchase behavior. Hong and Pavlou (2014) argued that product uncertainty (i.e., fit and quality) mediates the relationship between experience goods (i.e., products that have experiential attributes, such as clothes or shoes) and product returns/consumer satisfaction. Their findings underscored that sufficient product knowledge bolsters consumers’ familiarity with products, mitigates product uncertainty, and affects their product return behavior. Zhang et al. (2020) also asserted the mediating role of perceived uncertainty and psychological distance in the impact of live video streaming on online purchase intention. The authors found that live video streaming provides consumers with concrete product information based on two-way interaction and affects their decision-making by decreasing consumers’ perceived product uncertainty. In particular, when consumers shop for experiential goods (i.e., clothing, beauty products), product uncertainty is highly involved in their product assessment process, which causes them to evaluate products carefully before making a purchase decision (Workman, 2010). As livestream shopping provides product information through various touchpoints that deliver product information, it would reduce product uncertainty (e.g., Tang & Lin, 2019). The mitigated product uncertainty followed by information sources will ultimately explain purchase intention. Therefore, we suggest that product information sources achieved in livestream shopping would have positive indirect effects on purchase intention through the negative mediation of decreased product uncertainty. All hypotheses are presented in Fig. 1.

- H7. The three product information sources—(a) product demonstration, (b) interaction with the seller, and (c) other viewers’ reviews—will indirectly influence fashion consumers’ purchase intention through reduced product uncertainty in livestream shopping.

## Methods

### Data collection

After approval was obtained from the Institutional Review Board (IRB), the data were collected via an Amazon Mechanical Turk (MTurk) online survey. Participants were consumers 18 years or older who had livestream shopping experiences. A screening question was used to prevent those who had not experienced livestream shopping from participating in the survey. To verify their eligibility and ensure the quality of the data, they were asked about their recent livestream shopping experiences for purchasing fashion products. After incomplete responses were excluded, 292 usable surveys were used in further analysis. Approximately 63% of the participants were female and 36% were male. Most participants were from 25 to 44 years old (78%), Caucasian (70%), had a college-level education (63%), and a total household income of $25,000-$99,999 (73%). The fashion products the survey participants experienced through livestream shopping were apparel (30%), shoes (18%), accessories (14%), bags (12%), and others (6%).

### Measurement

A questionnaire was developed to measure two product information sources in physical store shopping, the three product information sources in livestream shopping, consumers’ uncertainty about fashion products, and their subsequent purchase intention. Items that asked about their brick-and-mortar store experiences were adapted from previous studies—need for touch (Citrin et al., 2003; α = 0.93) and salespeople’s assistance (Reynolds & Beatty, 1999; α = 0.90). Items in product demonstration (via passive strategy) (α = 0.80), interaction with the seller (via interactive strategy) (α = 0.86), and other viewers’ reviews (via active strategy) (α = 0.78) were adapted from previous studies and slightly modified to fit the livestream shopping context (Berger & Burgoon, 1995; Ou et al., 2014; Tang & Lin, 2019). Product uncertainty (α = 0.86) was measured by adopting the items from Dimoka et al. (2012), and purchase intention (α = 0.92) items were adopted from Hausman and Siekpe (2009). All items were measured on a 5-point Likert-type scale.

## Results

### Measurement model

Analysis of the partial least squares (PLS) model was conducted by assessing the measurement model and structural equation model. First, the measurement model was examined by evaluating the measurements’ reliability and the variables’ discriminant validity. The factor loadings of all items that were used for further analysis were ≥ 0.78, and the composite reliability for all constructs was ≥ 0.87, which indicated that all constructs were reliable. The Fornell-Larcker criterion indicated that the AVEs of the constructs were above 0.50, and the square root of the AVE was higher than the correlation between the two constructs (Fornell & Larcker, 1981), demonstrating that the measurement model of this study meets the discriminant validity requirement (see Table 1). The results obtained from the heterotrait-monotrait (HTMT) ratio of the correction technique do not exceed the threshold value of 0.90 (Hair et al., 2017) (see Table 2). Full collinearity variance inflation factors (VIF) were also assessed to check common method bias possibilities of the PLS-SEM, and all the VIF scores were below the threshold value of 3, confirming that the measurement model does not have a common bias (Kock, 2015).

**Table 1:**
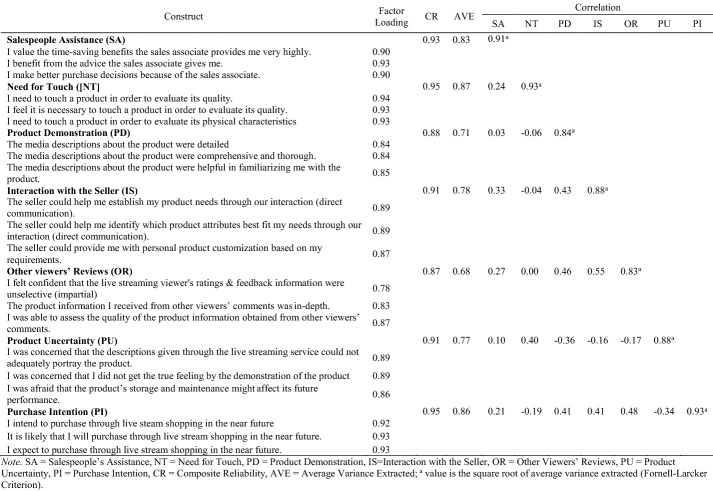
Measurement results

**Table 2:** Heterotrait–monotrait (HTMT) ratio

	SA	NT	PD	IS	OR	PU	PI
SA							
NT	0.257						
PD	0.056	0.070					
IS	0.380	0.061	0.520				
OR	0.319	0.092	0.580	0.682			
PU	0.114	0.442	0.426	0.188	0.203		
PI	0.230	0.203	0.477	0.457	0.564	0.386	

*SA* Salespeople’s Assistance, *NT* Need for Touch, *PD* Product Demonstration, *IS* Interaction with the Seller, *OR* Other Viewers’ Reviews, *PU* Product Uncertainty, *PI* Purchase Intention, The HTMT ratio also presents that all values do not exceed the threshold values of 0.90 (Hair et al., 2017)

### Structural model results

Partial least squares structural equation modeling (PLS-SEM) was conducted to test the research model and proposed hypotheses (see Fig. 1), and bootstrapping was used to estimate the precision of the PLS results and support the hypotheses (Fornell & Larcker, 1981) (see Table 3). The path coefficients confirmed that consumers who rely on obtaining product information from salespeople’s assistance (SA) at brick-and-mortar stores perceived a greater benefit of obtaining product information from interactions with the seller, and other viewers’ reviews (IS and OR) via the interactive and active communication strategies in livestream shopping, which supported H1b and H1c (H1b: β = 0.36, *p* < 0.001, H1c: β = 0.28, *p* < 0.001), while there was no significant path between salespeople’s assistance (SA) and the seller’s product demonstration (PD), rejecting H1a. The need for touch (NT) in brick-and-mortar store shopping and the three product information sources (PD, IS, and OR) in livestream shopping was not statistically significantly associated; thus, H2 was rejected. With respect to the relationship between a brick-and-mortar shopping experience and product uncertainty, the path coefficient confirmed that the need for touch (NT) reduces product uncertainty (PU) significantly, which supported H3b (β = 0.37, *p* < 0.001), while salespeople’s assistance (SA) had no significant influence on reducing product uncertainty (PU), which led to the rejection of H3a. In addition, the results indicated that the seller’s product demonstration (PD) in livestream shopping reduces product uncertainty (PU) significantly, and thus, H4a was supported (β = 0.32, *p* < 0.001). However, the interaction with the seller (IS) or other viewers’ reviews (OR) did not reduce uncertainty, and led to the rejection of H4b and H4c. Further, product uncertainty (PU) influenced purchase intention (PI) significantly and negatively, supporting H6 (β = − 0.22, *p* < 0.001). In terms of the direct effects of the three product information sources in livestream shopping on purchase intention, the interaction with the seller (IS) and other viewers’ reviews (OR) influenced consumers’ purchase intention (PI) positively, supporting H5b and H5c (H5b: β = 0.15, *p* < 0.05, H5c: β = 0.30, *p* < 0.001), while there was no significant direct influence of product demonstration (PD) on purchase intention (PI), rejecting H5a.

**Table 3:** Structural results

Paths	β	*t-*values	Confidence interval (95%)	VIF	*f*^2^
*Direct Path Results: H1–H6*					
H1a: SA → PD	0.048	0.691	[− 0.090; 0.191]	1.058	0.002
H1b: SA → IS	0.361^***^	6.121	[0.245; 0.474]	1.058	0.141
H1c: SA → OR	0.283^***^	4.300	[0.151; 0.406]	1.058	0.081
H2a: NT → PD	− 0.067	0.929	[− 0.206; 0.073]	1.058	0.004
H2b: NT → IS	− 0.120	1.914	[− 0.247; 0.003]	1.058	0.016
H2c: NT → OR	− 0.066	1.011	[− 0.194; 0.059]	1.058	0.004
H3a: SA → PU	0.030	0.508	[− 0.088; 0.144]	1.252	0.001
H3b: NT → PU	0.370^***^	6.683	[0.259; 0.476]	1.075	0.173
H4a PD → PU	− 0.323^***^	5.521	[− 0.439; − 0.211]	1.379	0.103
H4b IS → PU	− 0.007	0.093	[− 0.158; 0.143]	1.660	0.000
H4c: OR → PU	− 0.023	0.326	[− 0.162; 0.112]	1.603	0.000
H5a: PD → PI	0.129	1.760	[− 0.004; 0.285]	1.482	0.017
H5b: IS → PI	0.151^*^	2.124	[0.007; 0.286]	1.528	0.022
H5c: OR → PI	0.299^***^	3.751	[0.140; 0.456]	1.567	0.085
H6: PU → PI	− 0.221^***^	4.084	[− 0.321; − 0.111]	1.145	0.064
*Indirect Path Results: H7*					
H7a: PD → PU → PI	0.071^***^	3.635	[0.035; 0.111]		
H7b: IS → PU → PI	0.002	0.093	[− 0.033; 0.036]		
H7c: OR → PU → PI	0.005	0.321	[− 0.024; 0.039]		
*Explained variance*					
R^2^ PD	0.005				
R^2^ IS	0.125				
R^2^ OR	0.075				
R^2^ PU	0.269				
R^2^ PI	0.333				

*SA* Salespeople’s Assistance, *NT* Need for Touch, *PD* Product Demonstration, *IS* Interaction with the Seller, *OR* Other Viewers’ Reviews, *PU* Product Uncertainty, *PI* Purchase Intention

Significant at *p < .05, **p < .01, ***p < .001

With respect to the specific indirect effects based on bootstrapping procedures, coefficients were calculated by multiplying the corresponding paths, and the significance of the results was assessed using a bias-corrected CI. Bias corrected CI was obtained by adding bias (i.e., the difference between the estimated indirect effects from the path model and the mean value of the indirect effects from the bootstrap sample) to lower bound and upper bounds (Latan & Noonan, 2017). In the mediation analysis using PLS, the bias-corrected bootstrap confidence interval is an accurate approach to detect mediation effects (Hayes & Scharkow, 2013). The results of the three specific indirect effects of the model were obtained based on this approach indicated that the seller’s product demonstration (PD) in livestream shopping influenced consumers’ purchase intention (PI) positively mediated through product uncertainty reduction (PU) (H7a: β = 0.07, *p* < 0.001), which supported H7a, while the two other product information sources had no statistically significant indirect effects on purchase intention, rejecting H7b and H7c (see Table 3). The positive indirect effect of PD on PI (H7a) (β = 0.07, *p* < 0.001) is supported by multiplying the two negative direct effects from H4a (β = − 0.323) and H6 (β = − 0.221) and bias-corrected 95% CI (range of 0.035 to 0.111). Product demonstration (PD) negatively affects product uncertainty (PU), and product uncertainty (PU) negatively influences purchase intention (PI). In other words, product demonstration (PD) reduces product uncertainty (PU), and reduced product uncertainty (PU) leads to increased purchase intention (PI). Cohen’s effect sizes (*f*^2^) were also examined, and the effect sizes of the significant paths were all above 0.02 (Cohen, 1977).

## Discussion

This study shows that consumers who rely on salespeople’s assistance as a product information source showed positive perception toward the two sources—interaction with the seller and other viewers’ reviews—of obtaining product information in livestream shopping. This may be interpreted based on the livestream shopping features. Compared to the traditional online shopping format, livestream shopping allows consumers to engage in two-way real-time interactive communication that consumers perceive to be similar to the social interaction at brick-and-mortar stores. With the availability of real-time communication, consumers can receive instant feedback or answers to their questions about the product (Sun et al., 2019); further, they can learn about product features through interactions with other consumers over the live chat (Men & Zheng, 2019). Interestingly, however, we found a non-significant relation between salespeople’s assistance available at brick-and-mortar store shopping and the seller’s product demonstration in the livestream shopping context. This may be because the experience with livestream shopping services is sufficiently unique to distinguish the way consumers acquire product information from the traditional way of receiving assistance from salespeople. The livestream platform offers a vicarious experience for consumers; they observe sellers’ try-on and can envision the way that the sellers feel by watching their product demonstration through their tangible and psychological experience with the product (Chen et al., 2019). Further, streamers’ try-on and the demonstration of their experiences may help prevent sellers from omitting accurate information about the product and allow viewers to obtain unfiltered information through its real-time feature (Khobra & Gaur, 2020). Salespeople in brick-and-mortar stores do not offer this type of service. While salespeople’s service could be more related to interpersonal mechanisms, which help consumers be engaged in purchasing directly, seller’s product description through live streaming might be simply the source where consumers obtain product information. Therefore, consumers may differently perceive salespeople’s assistance in physical store shopping and product demonstration in livestream shopping, particularly when obtaining product information.

Moreover, the findings imply that consumers who rely on the touch and feel at brick-and-mortar stores do not have positive perception towards the three ways of acquiring product information in livestream shopping. This finding importantly indicates the uniqueness of brick-and-mortar shopping regarding consumer experiences with touching and trying-on products and also shows that such experiences may not be replaced with vicarious experiences through streamers in live stream shopping. Even though livestream shopping provides consumers with real-time experiences through streamers, the service has limitations to evaluate the product compared to brick-and-mortar shopping because the product evaluation requires one’s subjective experience through instrumental touch.

With regard to the influence of two information sources of brick-and-mortar store shopping on product uncertainty, the need for touch has significantly reduced product uncertainty while salespeople’s assistance did not show a significant effect. This indicates that consumers might not perceive salespeople’s assistance as a powerful information source at brick-and-mortar stores. Although previous researchers found that salespeople’s assistance could guide consumers and help consumers navigate stores and find products (Haas & Kenning, 2014), it may not be significantly directed to lower consumers’ uncertainty toward products at brick-and-mortar stores. One plausible reason for this is that salespeople’s assistance helps consumers’ decision-making through consumers’ affect or emotions and satisfaction with their shopping experience (Goff et al., 1997). In other words, consumers may tend to get help from salespeople for different purposes rather than obtaining product information, such as social interaction or engagement with the store. In this case, consulting with salespeople may induce their positive attitude toward brick-and-mortar store shopping but not contribute to expanding their product knowledge.

Livestream shopping’s direct effects on product uncertainty and purchase intention showed various patterns. Product demonstration played a significant role in reducing product uncertainty, while the other two information sources (i.e., interaction with the seller and other viewers’ reviews) did not significantly influence. However, interaction with the seller significantly affected the purchase intention, while product demonstration and other viewers’ reviews did not have an effect. This is because consumers perceive product demonstrations as information sources to obtain product information and reduce the level of their product uncertainty. Product demonstration may not prompt consumers’ purchase intention directly; rather, consumers’ purchase intention is induced by interaction with the seller through real-time communication. It can be explained by the two different consumers’ perceptions, cognitive versus affective. When consumers view the seller’s product demonstration, they perceive the information based on their cognitive perception. However, interaction with the seller or other viewers’ reviews may affect consumers’ purchase decisions through their emotions, and these two sources are not engaged with the further information-related process, which is uncertainty reduction. This also implies that there might be two or more different mechanisms to increase consumers’ purchase intention in a livestream shopping format. One is through product uncertainty reduction, and the other is through some other aspects of livestream shopping, such as trust in the seller or social ties with the seller. Overall, the study confirmed the previous studies that examined the significant role of live streaming’s product presentation in reducing uncertainty in e-commerce (Chen et al., 2019). Additionally, the findings of this study extend previous livestream studies by connecting brick-and-mortar and livestream shopping experiences. This study compared consumers’ perceptions of product information sources used in brick-and-mortar store shopping and the ones used in livestream shopping and found two different mechanisms that lead to consumers’ purchase decisions. This study provides that livestream shopping’s real-time feature can influence consumers’ cognitive aspect of decision-making by its enhanced transparency of functional and practical information about products, which reduces information asymmetry between consumers and sellers.

With regard to the indirect effects of livestream shopping on purchase intention, importantly, we found a significant indirect influence of product demonstration on consumers’ purchase intention mediated by reduced product uncertainty, while the other two sources, interaction with the seller and other viewers’ reviews, did not have indirect relationships with purchase intention through reduced product uncertainty. Product uncertainty derives more likely from utilitarian shopping values. Thus, it can be explained that consumers are inclined to obtain details about a product through the seller’s product demonstration to reduce product uncertainty because their utilitarian shopping values are not fulfilled in the livestream shopping environment, as they are separated from the product physically. Perhaps the other two sources (i.e., interaction with the seller and other viewers’ reviews) are more likely to be associated with purchase intention through different routes more related to hedonic shopping values or social elements of live streaming features that are not necessarily related to product uncertainty. While prior livestream shopping-related studies were highly focused on hedonic benefits, this study revealed the utilitarian benefits of livestream shopping by finding the cognitive aspects of consumers’ perceptions that led to their purchase decision.

## Conclusions

### Theoretical implications

The results of this study provide threefold theoretical implications. First, the findings extend the URT to improve our understanding of consumer information attainment in the livestream shopping context. While previous studies have adopted the URT for e-commerce shopping behavior and information disclosure behavior in the social media context (Cai & Wohn, 2019; Sun et al., 2019; Wongkitrungrueng & Assarut, 2020), the study expands the application of URT to the livestream shopping context. Within the URT, this study attempts to adopt three strategies of product information acquisition and redefine them within the livestream shopping context, which provides scholastic implications. This is because such academic effort undertakes the importance of understanding the add-on live streaming service as an effective and emerging service tool that alleviates product uncertainty, ultimately increasing purchase intention.

Second, this study contributes to developing a framework by bridging consumers’ sources of product information at physical stores with those at livestream shopping. This attempt would provide an understanding of the connection between consumers’ expectations toward product information sources from brick-and-mortar store shopping to livestream shopping. By exploring various sources of product information in brick-and-mortar store shopping (i.e., salespeople’s assistant, need for touch) and livestream shopping (i.e., product demonstration, interaction with the seller, other viewers’ review), this study examined the relationship between these two shopping formats focusing on obtaining product information from a consumer’s perspective. This study exemplifies the promising future of livestream shopping by investigating its features that can potentially substitute for the brick-and-mortar shopping experience.

Finally, this study provides a practical perspective on livestream shopping. For example, our results shed light on the utilitarian benefit of livestream fashion shopping (i.e., accessing product information), while previous livestream studies have focused on the hedonic perspective that can benefit consumers by offering them an enjoyable and entertaining experience attributable to the use of novel technical features (Cai & Wohn, 2019; Sun et al., 2019; Wongkitrungrueng & Assarut, 2020). Thus, this study provides theoretical approaches that explain livestream shoppers’ behavior by emphasizing the role of livestream services in reducing product uncertainty and increasing purchase intention from a product information acquisition perspective.

### Managerial implications

This study provides several managerial implications. First, unlike previous literature that focused on the hedonic benefits obtained from real-time interactions and authentic technical features in livestreaming services, this study provides fashion e-retailers with a practical lens through which to understand livestreaming’s role in reducing product uncertainty in online shopping. Particularly, product demonstrations should be incorporated actively into fashion retailers’ livestreaming services to reduce product uncertainty. The seller’s demonstration of product features significantly reduced product uncertainty in livestream shopping in this study, implying that live streaming services can benefit fashion consumers who use this for utilitarian purposes (i.e., obtaining or increasing product knowledge). Fashion e-retailers should consider providing extensive streaming tactics to deliver product information by allocating more time and educating streamers to boost their abilities to demonstrate products. Further, they can add additional functions to live streaming services to allow audiences (i.e., potential consumers) to click and expand the product details.

Second, this study identifies the way consumers’ motivations that lead them to be engaged more in offline shopping can be associated with the benefits of livestream shopping. According to our results, consumers who rely on salespeople’s assistance in physical retail stores positively perceived interaction with the seller and other viewers’ reviews in livestream shopping for obtaining product information. This provides implications to store-based retailers that live streaming services’ interactive features, such as providing accessible real-time information (e.g., others’ reviews) and one-on-one interactions (e.g., between audiences and the seller), are critical to encouraging offline consumers to try livestream shopping. Although the interaction with the sellers and other viewers’ reviews did not reduce consumers’ product uncertainty directly, they are still essential features in livestreaming services, as they increase product sales eventually.

Finally, fashion consumers’ need for touch and feel is the crucial and unique element of evaluating products in physical store shopping that livestream shopping benefits can neither compensate for nor replace. To satisfy fashion consumers’ demands for experiential and sensory benefits in e-commerce environments, retailers can consider adopting innovative haptic technologies that incorporate immersive try-on services or augmented reality technology. Kim and Ha (2019) discussed the importance of haptic communication technology in immersive virtual reality fashion shopping, as such technology can enhance the immersion experience and provide a sense of presence. They pointed out that fashion product shopping requires multi-sensory information compared to other product categories, and delivering a vivid tactile sense of products is a significant element that can provide an indulgent experience in an immersive virtual shopping environment.

### Limitations and future studies

Although this study developed a research model and collected the data carefully, the model did not attempt to specify other potential factors that may influence consumers’ product uncertainty and purchase intention during livestream fashion shopping. We examined a modest number of factors related to fashion consumers’ motivation to engage in brick-and-mortar stores and livestream shopping based on the URT by focusing on obtaining product information. Thus, to extend the model and increase its validity, future researchers may examine different motivational factors (i.e., service and/or technical barriers) related to services within the live streaming interface that can influence consumers’ purchase decisions.

## Data Availability

Please contact the authors for data requests.
